# Supplementary material for: Are adversities and worries during the COVID-19 pandemic related to sleep quality? Longitudinal analyses of 46,000 UK adults
Source: PLoS One. 2021 Mar 25;16(3):e0248919. doi: 10.1371/journal.pone.0248919 (PMC7993810; doi:10.1371/journal.pone.0248919)
Supplement: S1 Table — (DOCX) [file pone.0248919.s001.docx]

| Original | Adapted for COVID-19  In the past week, I feel… |
| --- | --- |
| I experience a lot of understanding and security from others | I have experienced a lot of understanding and support from others |
| I know a very close person whose help I can always count on | I have a very close person whose help I can always count on |
| If necessary, I can easily borrow something I might need from neighbours or friends | If necessary, I can easily borrow something I need from neighbours or friends |
| I know several people with whom I like to do things | I have people with whom I can spend time and do things together |
| When I am sick, I can without hesitation ask friends and family to take care of  important matters for me | If I get sick, I have friends and family who will take care of me |
| If I am down, I know to whom I can go without hesitation | If I am feeling down, I have people I can talk to without hesitation |

**S1 Table. Comparison of items in the original and revised perceived social support questionnaire (F-SozU K-6).**
