# Supplementary material for: Are adversities and worries during the COVID-19 pandemic related to sleep quality? Longitudinal analyses of 46,000 UK adults
Source: PLoS One. 2021 Mar 25;16(3):e0248919. doi: 10.1371/journal.pone.0248919 (PMC7993810; doi:10.1371/journal.pone.0248919)
Supplement: S2 Table — Weighted figures. (DOCX) [file pone.0248919.s002.docx]

|  | Variable | Mean (SD) / n (%) | Sleep Quality ρ / Mean (SD) |
| --- | --- | --- | --- |
|  | Social Support (F-SozU K-6) | 21.86 (6.11) | 0.23 |
|  | UCLA Loneliness | 4.91 (1.95) | -0.31 |
|  | Number of close friends | 4.53 (3.13) | 0.11 |
| Living Arrangement | Lives with others | 37,353.4 (80.7%) | 3.11 (1.11) |
|  | Lives alone | 8,930.6 (19.3%) | 3.02 (1.13) |
| Diagnosed with mental health condition | No illness | 37,549 (81.13%) | 3.22 (1.07) |
|  | Diagnosed illness | 8,735 (18.87%) | 2.56 (1.12) |

**S2 Table. Descriptive statistics, social support and psychiatric diagnosis.** Weighted figures.
