# Supplementary material for: Are adversities and worries during the COVID-19 pandemic related to sleep quality? Longitudinal analyses of 46,000 UK adults
Source: PLoS One. 2021 Mar 25;16(3):e0248919. doi: 10.1371/journal.pone.0248919 (PMC7993810; doi:10.1371/journal.pone.0248919)
Supplement: S3 Table — (DOCX) [file pone.0248919.s003.docx]

|  | Variable | Unweighted Data | Weighted Data |
| --- | --- | --- | --- |
| Age (grouped) | 18-34 | 6,920 (14.95%) | 8,811.41 (19.04%) |
|  | 35-49 | 13,858 (29.94%) | 11,475.51 (24.79%) |
|  | 50-64 | 15,651 (33.82%) | 14,509.37 (31.35%) |
|  | 65+ | 9,855 (21.29%) | 11,487.70 (24.82%) |
| Country of residence | England | 36,739 (79.38%) | 38,573.30 (83.34%) |
|  | Scotland | 3,126 (6.75%) | 3,901.46 (8.43%) |
|  | Wales | 5,926 (12.8%) | 2,789.89 (6.03%) |
|  | Northern Ireland | 493 (1.07%) | 1,019.34 (2.2%) |
| Mental health condition | No illness | 37,793 (81.65%) | 37,549.00 (81.13%) |
|  | Diagnosed illness | 8,491 (18.35%) | 8,735.00 (18.87%) |
| Physical health condition | No illness | 28,963 (62.58%) | 27,603.61 (59.64%) |
|  | Diagnosed illness | 17,321 (37.42%) | 18,680.39 (40.36%) |
| Highest qualification | None | 1,187 (2.56%) | 2,943.00 (6.36%) |
|  | GCSEs | 5,165 (11.16%) | 11,605.56 (25.07%) |
|  | Post-16 Vocational | 2,567 (5.55%) | 4,395.68 (9.5%) |
|  | A-Levels | 5,568 (12.03%) | 10,878.24 (23.5%) |
|  | Undergraduate degree | 19,089 (41.24%) | 9,862.25 (21.31%) |
|  | Postgraduate degree | 12,708 (27.46%) | 6,599.27 (14.26%) |
| Ethnicity | White | 44,219 (95.54%) | 41,840.13 (90.4%) |
|  | Non-White | 2,065 (4.46%) | 4,443.87 (9.6%) |
| Gender | Male | 11,584 (25.03%) | 22,524.33 (48.67%) |
|  | Female | 34,700 (74.97%) | 23,759.67 (51.33%) |
| Income (grouped) | < £16k | 6,048 (14.38%) | 8,350.57 (20.1%) |
|  | £16k - £30k | 10,077 (23.97%) | 11,484.37 (27.64%) |
|  | £30k - £60k | 14,772 (35.13%) | 13,436.50 (32.34%) |
|  | £60k - £90k | 6,578 (15.64%) | 5,008.57 (12.05%) |
|  | £90k+ | 4,571 (10.87%) | 3,271.75 (7.87%) |
| Marital status | Single | 7,316 (15.81%) | 8,997.68 (19.44%) |
|  | Divorced/Widowed | 6,168 (13.33%) | 5,968.86 (12.9%) |
|  | In relationship but living apart | 2,853 (6.16%) | 3,189.69 (6.89%) |
|  | Cohabiting with partner | 29,947 (64.7%) | 28,127.77 (60.77%) |

**S3 Table. Sample descriptive statistics.**
