# Supplementary material for: Are adversities and worries during the COVID-19 pandemic related to sleep quality? Longitudinal analyses of 46,000 UK adults
Source: PLoS One. 2021 Mar 25;16(3):e0248919. doi: 10.1371/journal.pone.0248919 (PMC7993810; doi:10.1371/journal.pone.0248919)
Supplement: S4 Table — (DOCX) [file pone.0248919.s004.docx]

| Week | n | % Last Interview |
| --- | --- | --- |
| 1 | 26,108 | 0% |
| 2 | 34,270 | 4.93% |
| 3 | 35,740 | 8.11% |
| 4 | 34,820 | 9.11% |
| 5 | 35,107 | 7.2% |
| 6 | 32,564 | 6.92% |

**S4 Table. Sample size by week.**
