# Supplementary material for: Are adversities and worries during the COVID-19 pandemic related to sleep quality? Longitudinal analyses of 46,000 UK adults
Source: PLoS One. 2021 Mar 25;16(3):e0248919. doi: 10.1371/journal.pone.0248919 (PMC7993810; doi:10.1371/journal.pone.0248919)
Supplement: S5 Table — (DOCX) [file pone.0248919.s005.docx]

| Outcome | Modifier | Adversity Experiences | Adversity Worries |
| --- | --- | --- | --- |
| Sleep quality | Social Support (F-SozU K-6) | -0.001 [-0.018, 0.016] | 0.001 [-0.006, 0.008] |
|  | UCLA Loneliness | 0.008 [-0.011, 0.027] | 0.002 [-0.005, 0.009] |
|  | Number of close friends | -0.015 [-0.035, 0.006] | 0.003 [-0.004, 0.01] |
|  | Lives alone | 0.012 [-0.036, 0.061] | 0 [-0.019, 0.02] |
|  | Diagnosed illness | -0.029 [-0.07, 0.012] | -0.002 [-0.018, 0.014] |
| Bad Sleep (Random Effects Logit) | Social Support (F-SozU K-6) | 1.016 [0.968, 1.065] | 0.986 [0.965, 1.007] |
|  | UCLA Loneliness | 0.952 [0.909, 0.998] | 0.987 [0.967, 1.007] |
|  | Number of close friends | 1.032 [0.98, 1.086] | 0.969 [0.948, 0.99] |
|  | Lives alone | 0.987 [0.875, 1.113] | 1.012 [0.96, 1.066] |
|  | Diagnosed illness | 1.104 [0.994, 1.227] | 1.014 [0.968, 1.063] |
| Bad Sleep (Linear Probability) | Social Support (F-SozU K-6) | 0.001 [-0.007, 0.009] | -0.002 [-0.006, 0.001] |
|  | UCLA Loneliness | -0.004 [-0.013, 0.005] | 0 [-0.003, 0.004] |
|  | Number of close friends | 0.003 [-0.006, 0.013] | -0.004 [-0.008, 0] |
|  | Lives alone | -0.002 [-0.024, 0.021] | 0.002 [-0.008, 0.011] |
|  | Diagnosed illness | 0.012 [-0.007, 0.032] | 0.004 [-0.004, 0.013] |
| Bad Sleep (Fixed Effects Logit) | Social Support (F-SozU K-6) | 1.013 [0.967, 1.063] | 0.984 [0.963, 1.004] |
|  | UCLA Loneliness | 0.957 [0.914, 1.002] | 0.986 [0.967, 1.006] |
|  | Number of close friends | 1.024 [0.973, 1.076] | 0.967 [0.947, 0.987] |
|  | Lives alone | 0.997 [0.884, 1.124] | 1.017 [0.965, 1.072] |
|  | Diagnosed illness | 1.1 [0.992, 1.22] | 1.008 [0.963, 1.055] |
| Bad Sleep (Random Effects Logit, Changers Only) | Social Support (F-SozU K-6) | 1.015 [0.966, 1.065] | 0.983 [0.963, 1.005] |
|  | UCLA Loneliness | 0.955 [0.911, 1.002] | 0.986 [0.966, 1.006] |
|  | Number of close friends | 1.026 [0.974, 1.081] | 0.966 [0.945, 0.987] |
|  | Lives alone | 0.993 [0.877, 1.123] | 1.014 [0.961, 1.07] |
|  | Diagnosed illness | 1.11 [0.997, 1.235] | 1.012 [0.965, 1.061] |

**S5 Table. Estimated interaction effects between social support and diagnosed psychiatric illness and deviation in total adversity experiences and adversity worries.**
