# Supplementary figures and images for: Are adversities and worries during the COVID-19 pandemic related to sleep quality? Longitudinal analyses of 46,000 UK adults
Source: PLoS One. 2021 Mar 25;16(3):e0248919. doi: 10.1371/journal.pone.0248919 (PMC7993810; doi:10.1371/journal.pone.0248919)

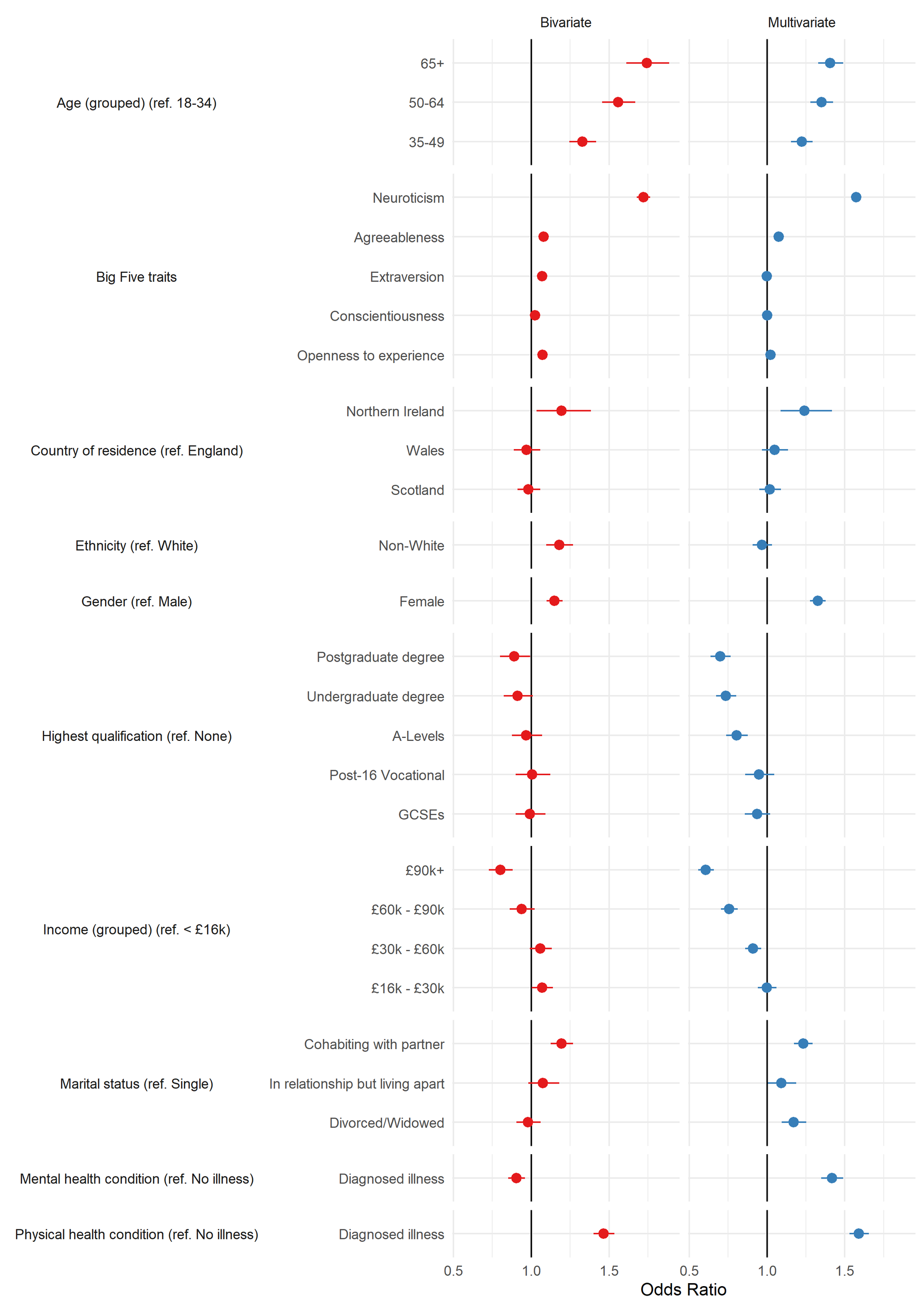

Supplement: S1 Fig — Derived from bivariate and multivariate survey weighted logistic regression models. Multivariate models include adjustment for each factor simultaneously. Big-Five personality traits are scaled (mean = 0, SD = 1). (TIF) [file pone.0248919.s006.tif]

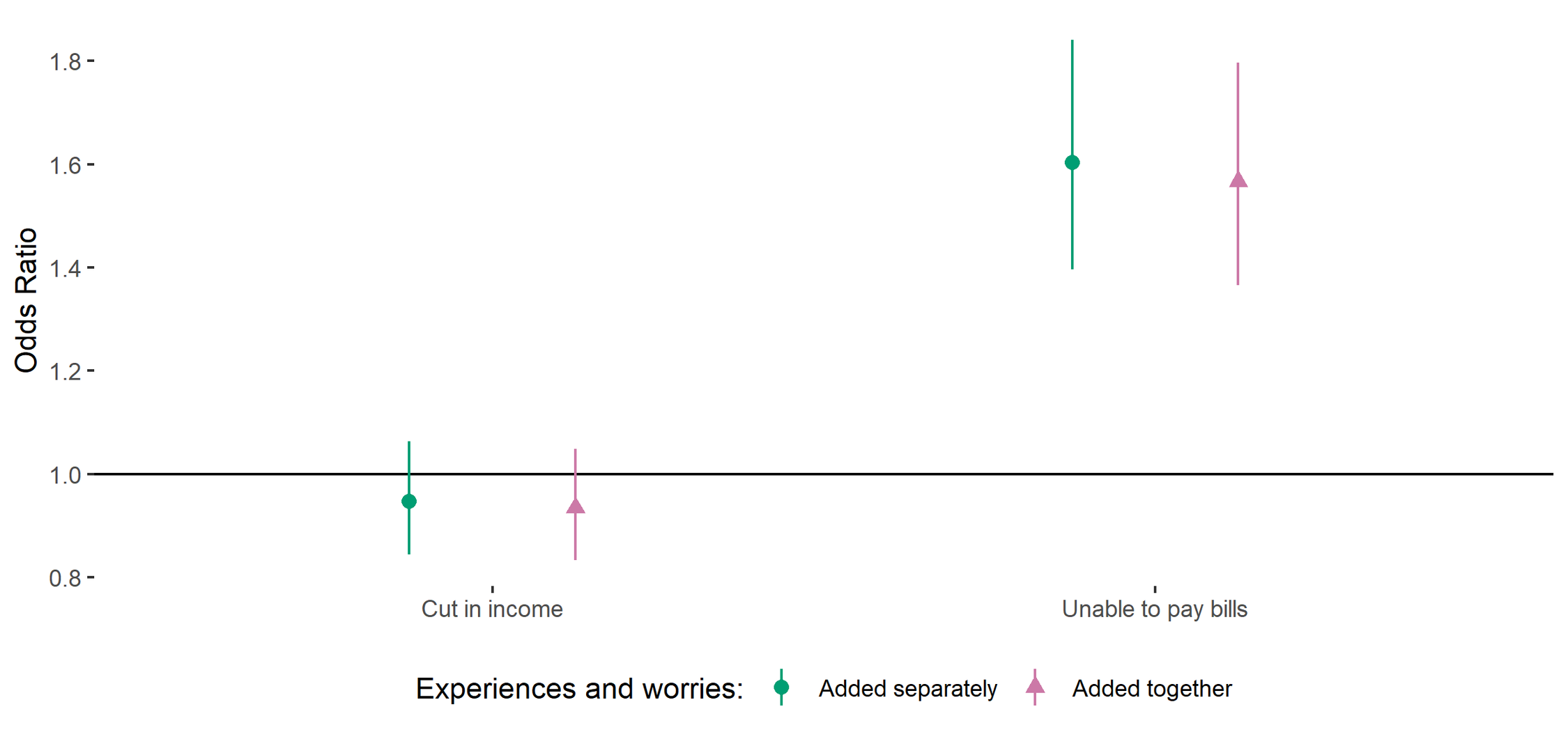

Supplement: S2 Fig — Derived from REWB models. (TIF) [file pone.0248919.s007.tif]

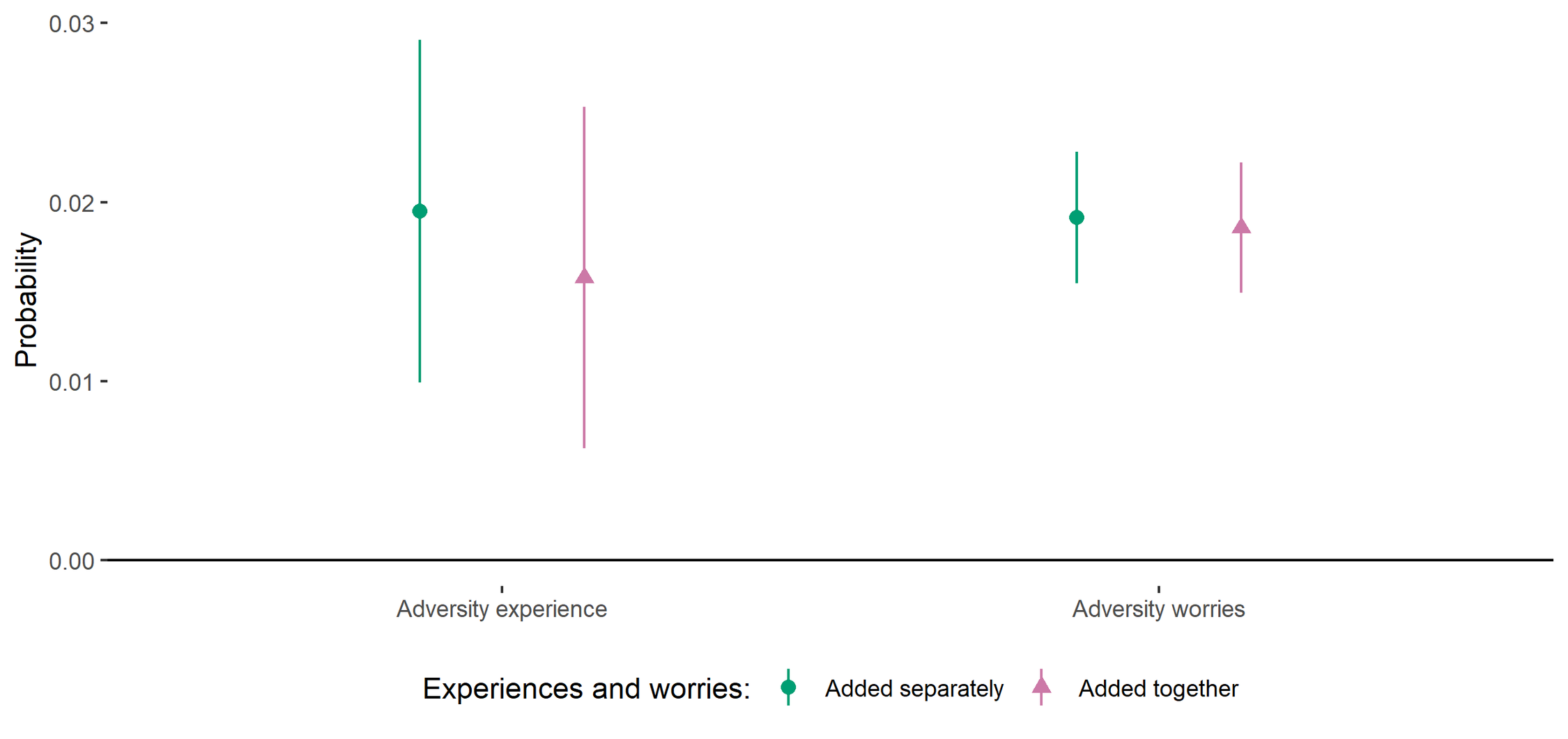

Supplement: S3 Fig — Derived from linear probability fixed effects models. (TIF) [file pone.0248919.s008.tif]

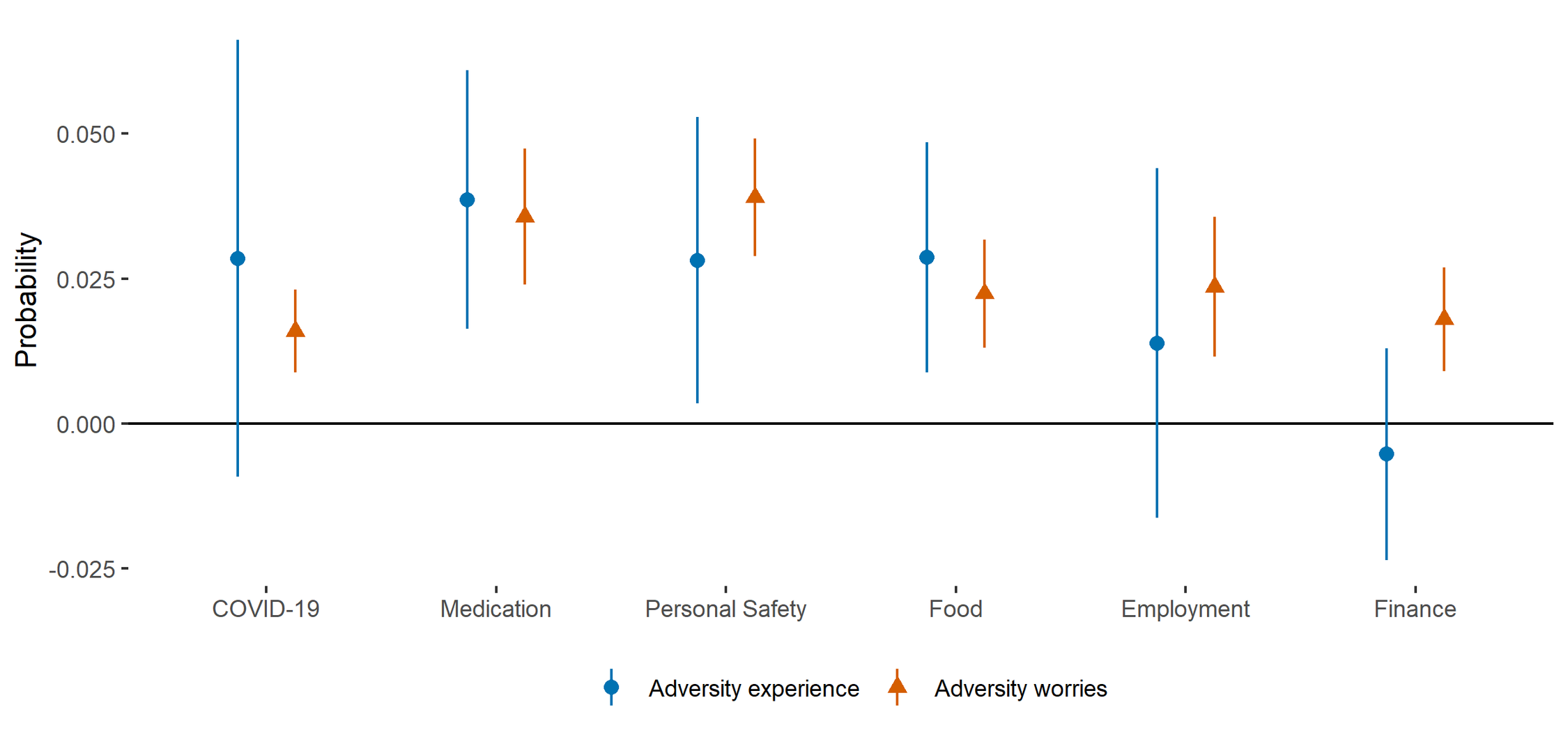

Supplement: S4 Fig — Derived from linear probability fixed effects models. (TIF) [file pone.0248919.s009.tif]

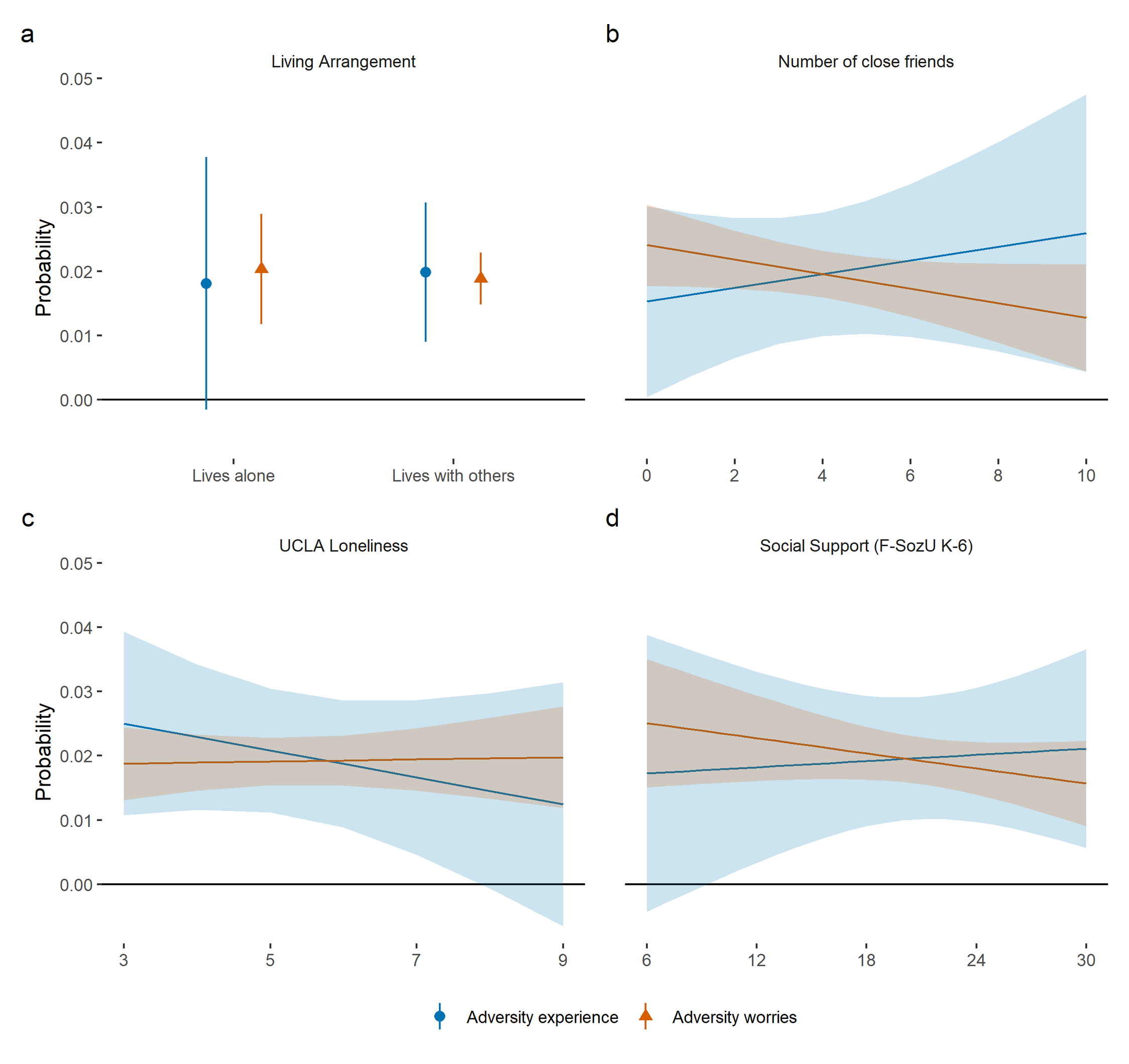

Supplement: S5 Fig — Derived from linear probability fixed effects models. (TIF) [file pone.0248919.s010.tif]

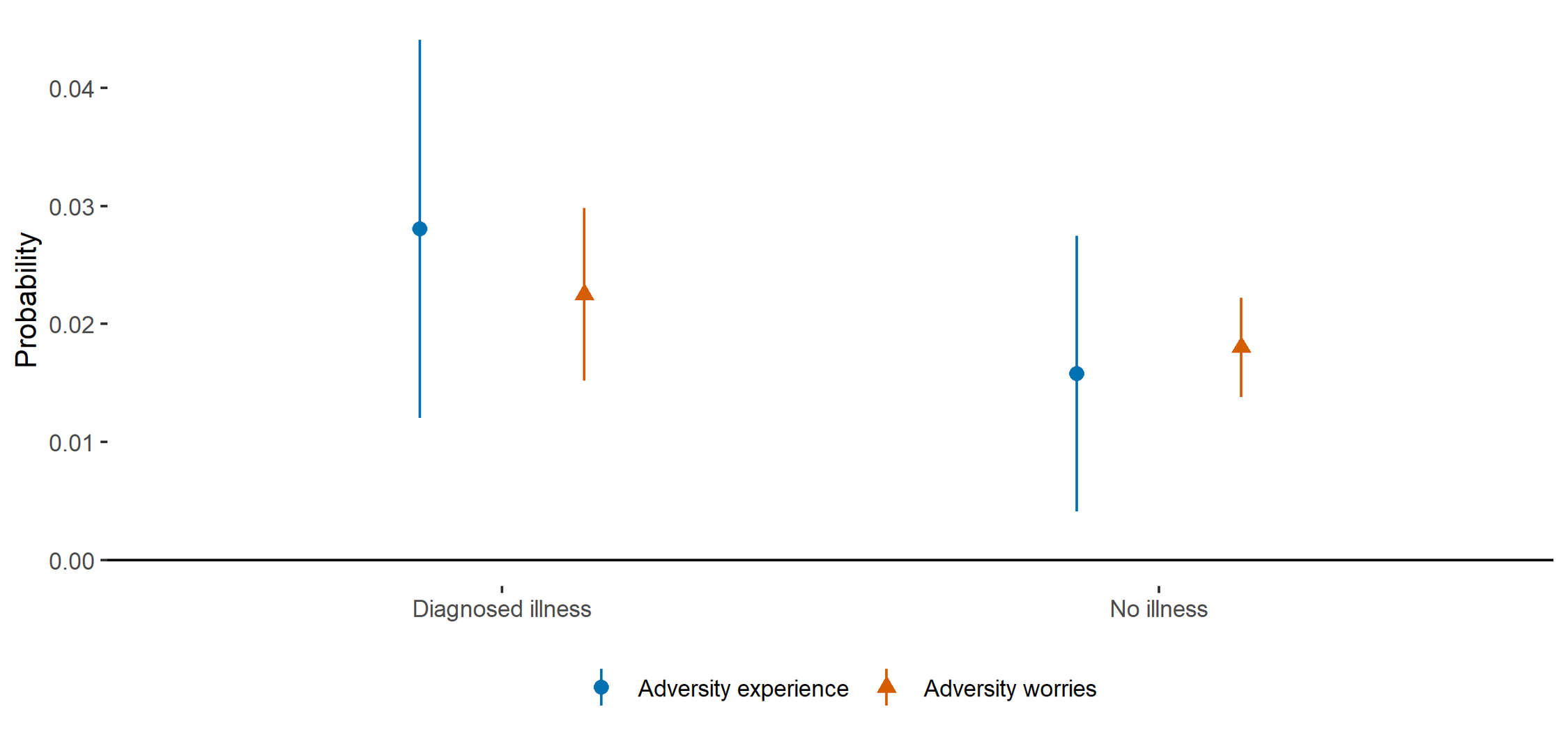

Supplement: S6 Fig — Derived from linear probability fixed effects models. (TIF) [file pone.0248919.s011.tif]

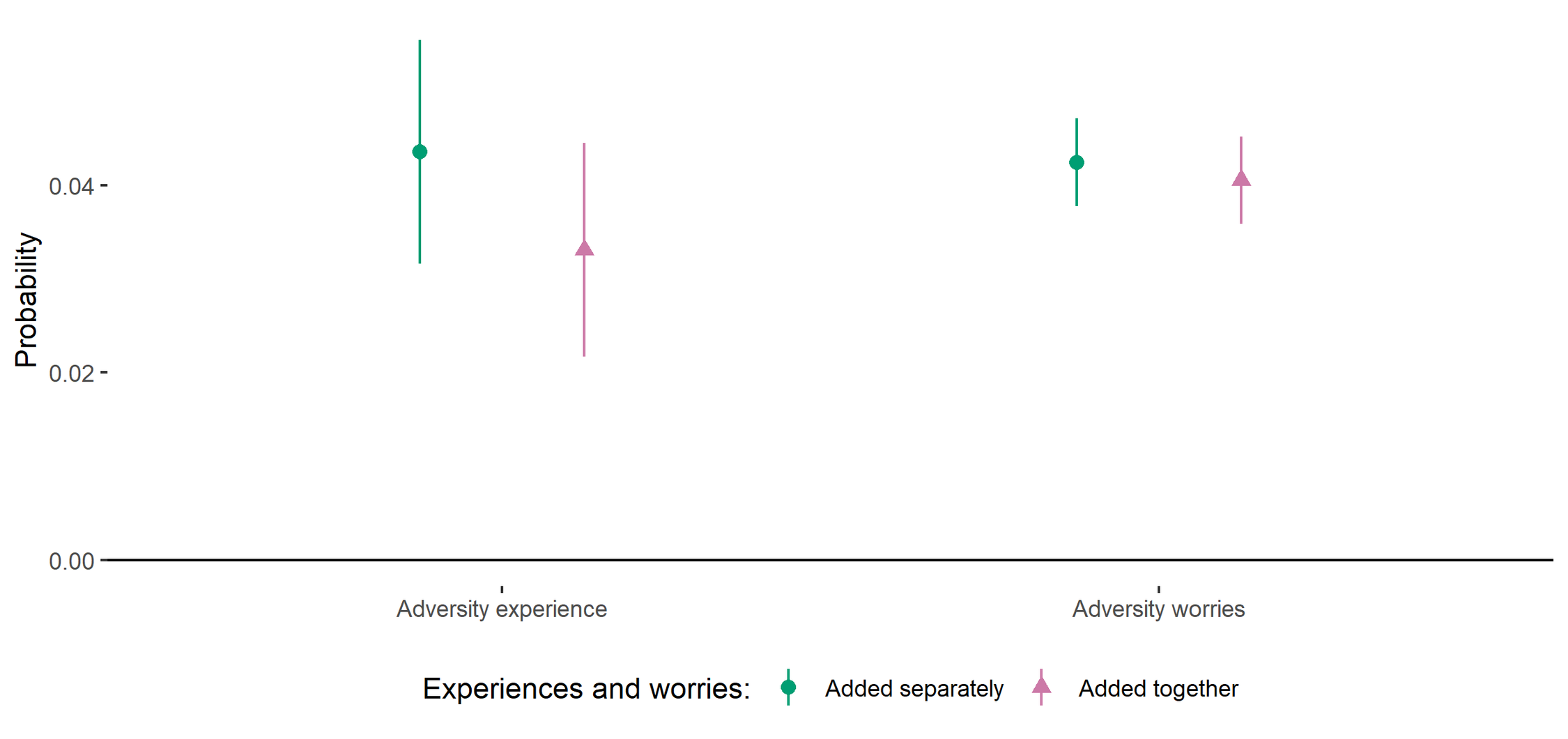

Supplement: S7 Fig — Derived from fixed effects logit models. (TIF) [file pone.0248919.s012.tif]

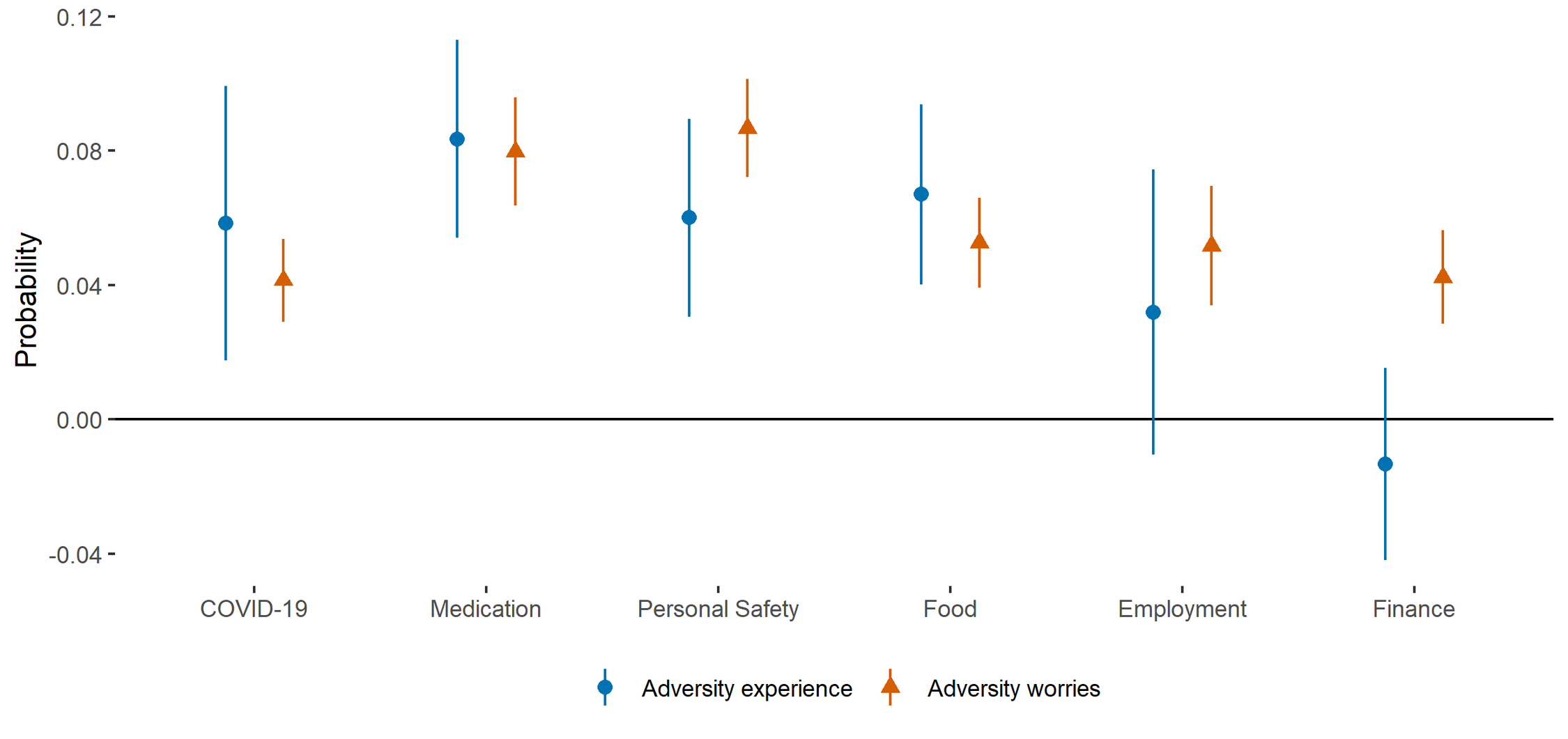

Supplement: S8 Fig — Derived from fixed effects logit models. (TIF) [file pone.0248919.s013.tif]

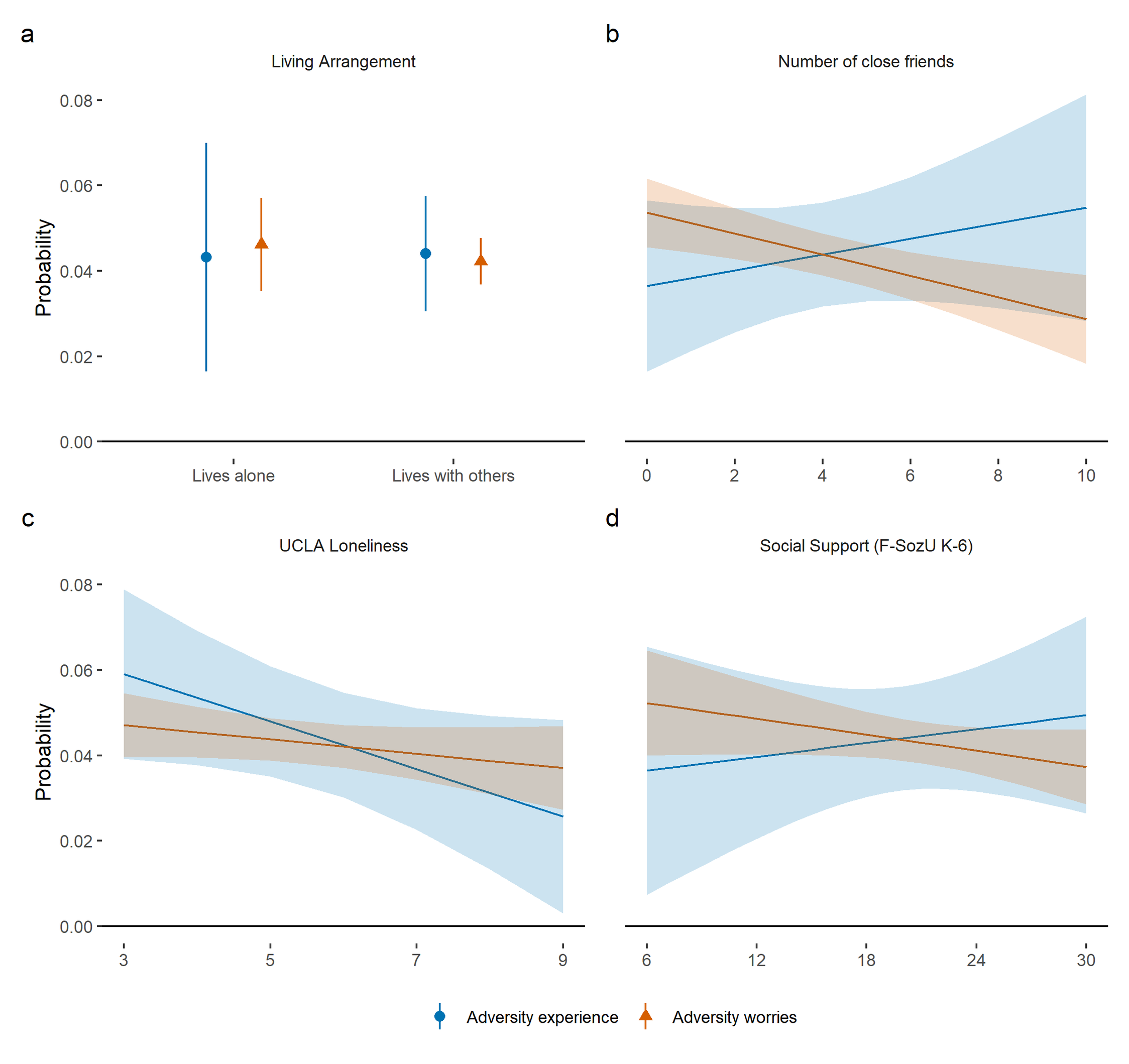

Supplement: S9 Fig — Derived from fixed effects logit models. (TIF) [file pone.0248919.s014.tif]

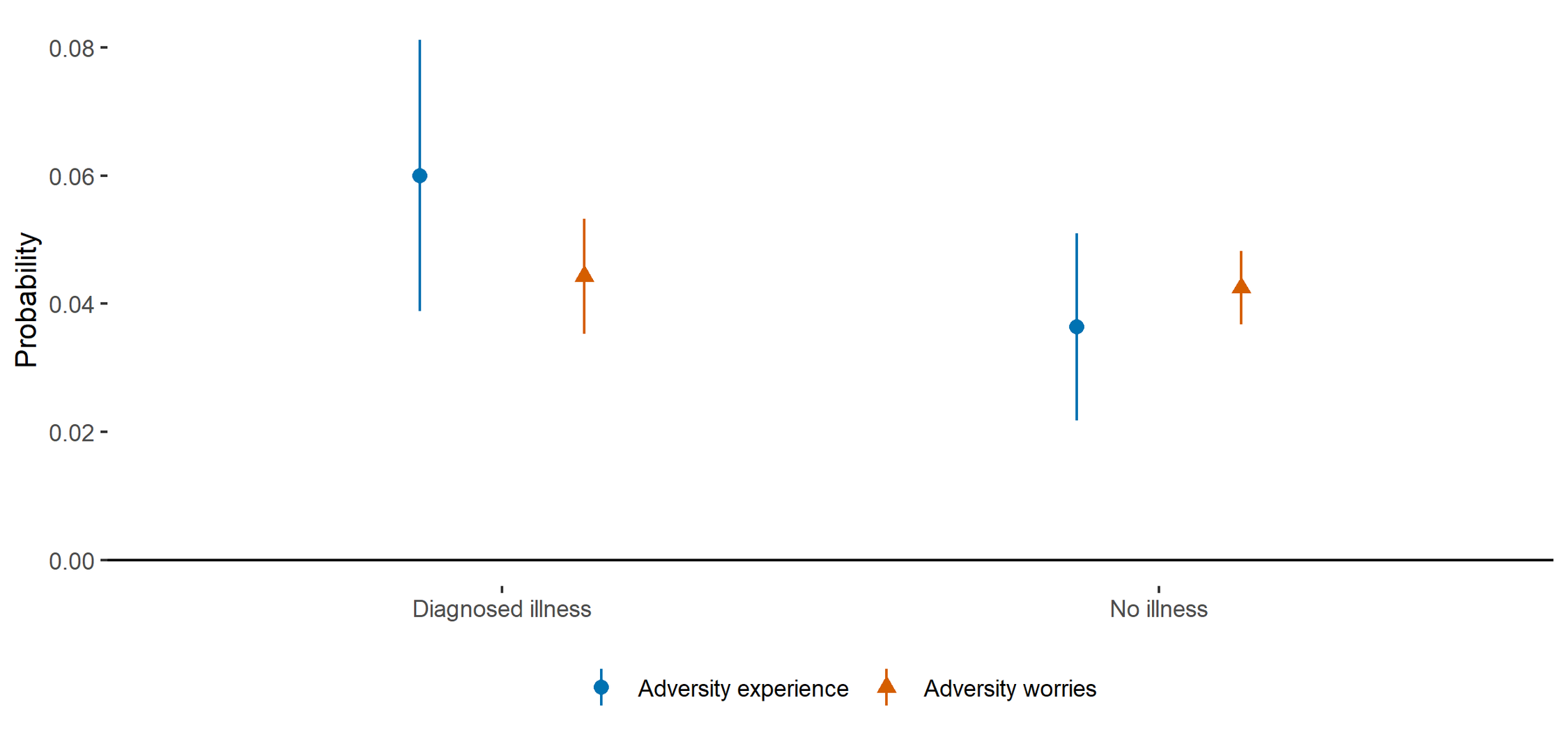

Supplement: S10 Fig — Derived from fixed effects logit models. (TIF) [file pone.0248919.s015.tif]

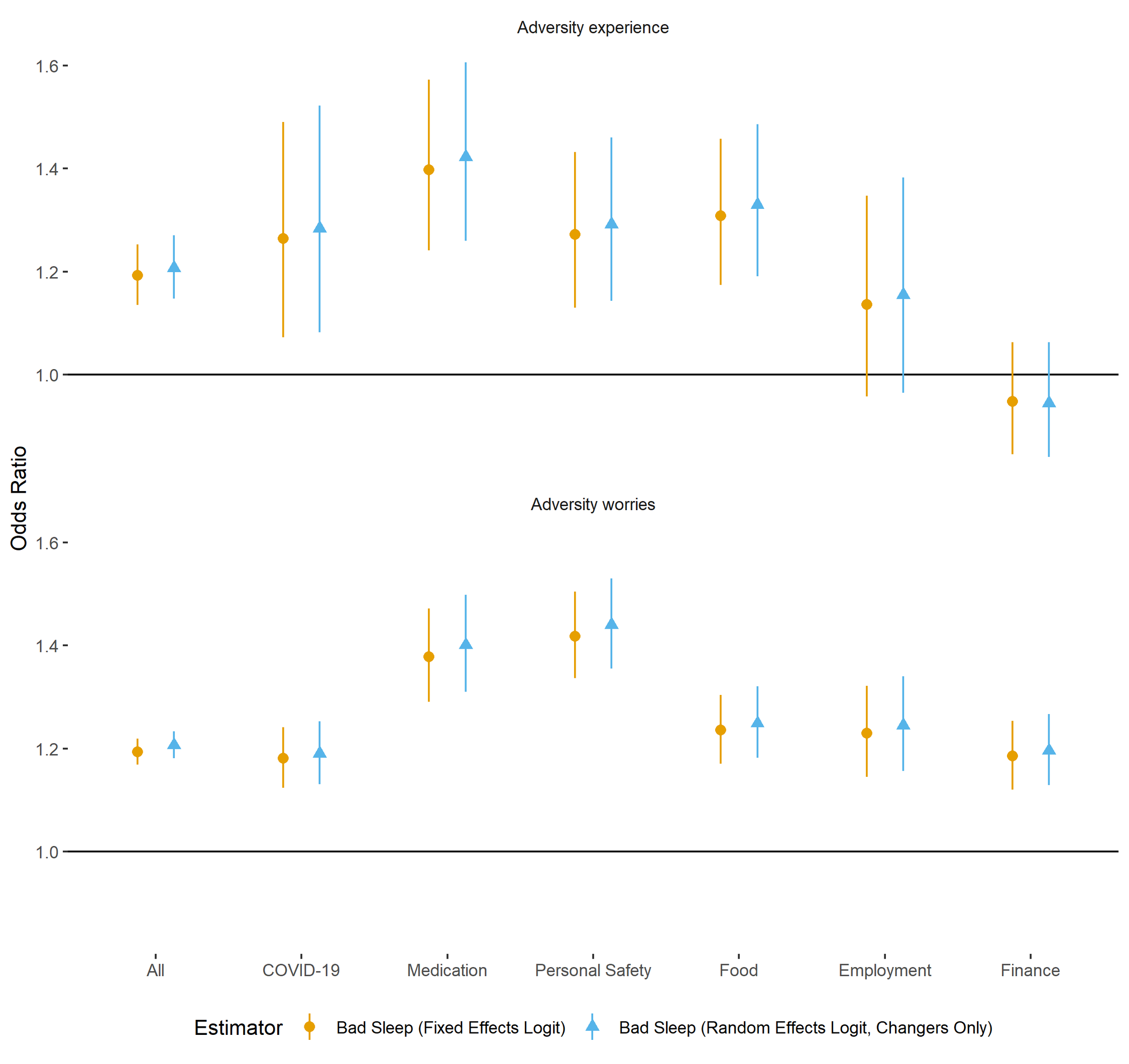

Supplement: S11 Fig — Derived from fixed effects logit models and REWB models, where sample is those whose sleep quality changed during follow-up period. (TIF) [file pone.0248919.s016.tif]

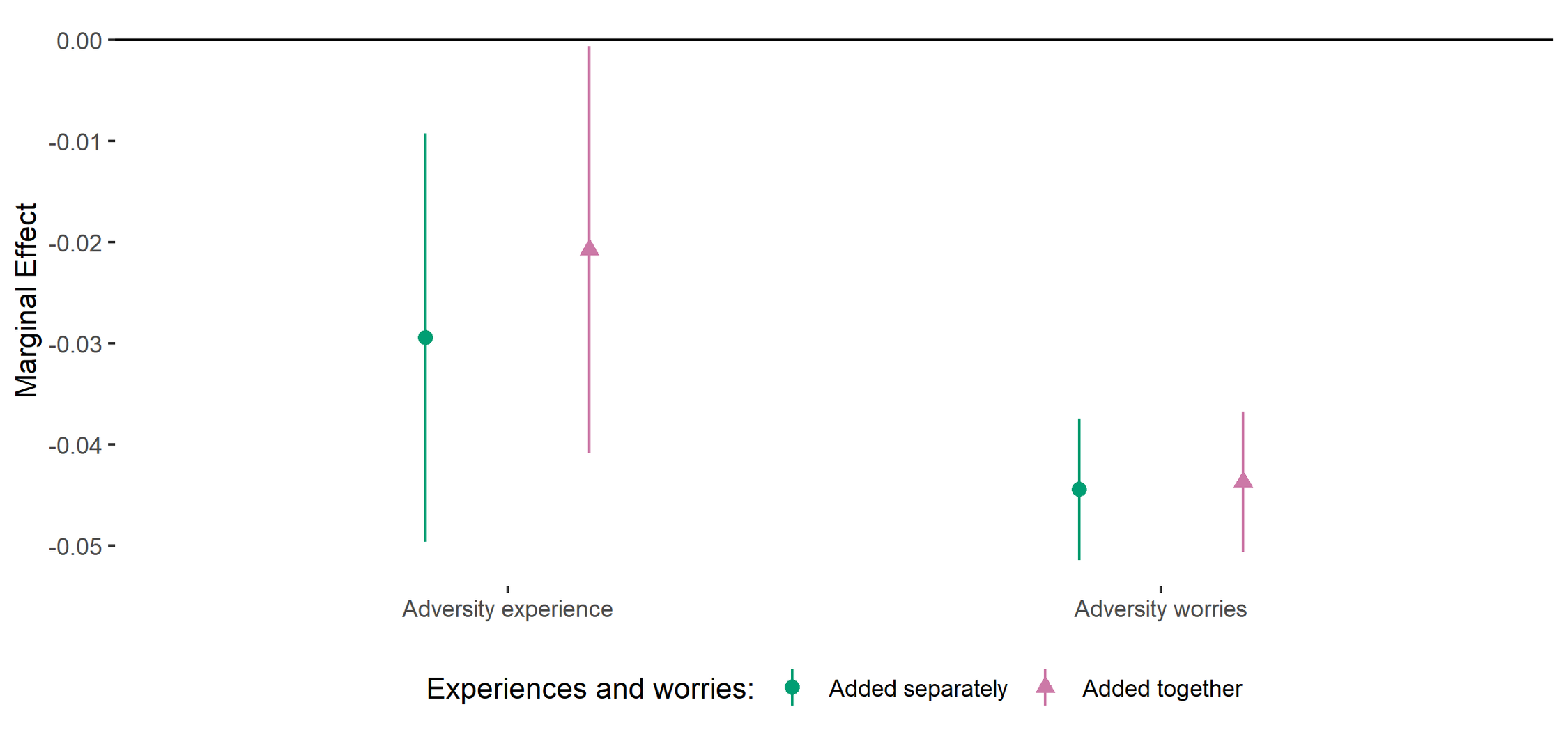

Supplement: S12 Fig — Derived from fixed effects models. (TIF) [file pone.0248919.s017.tif]

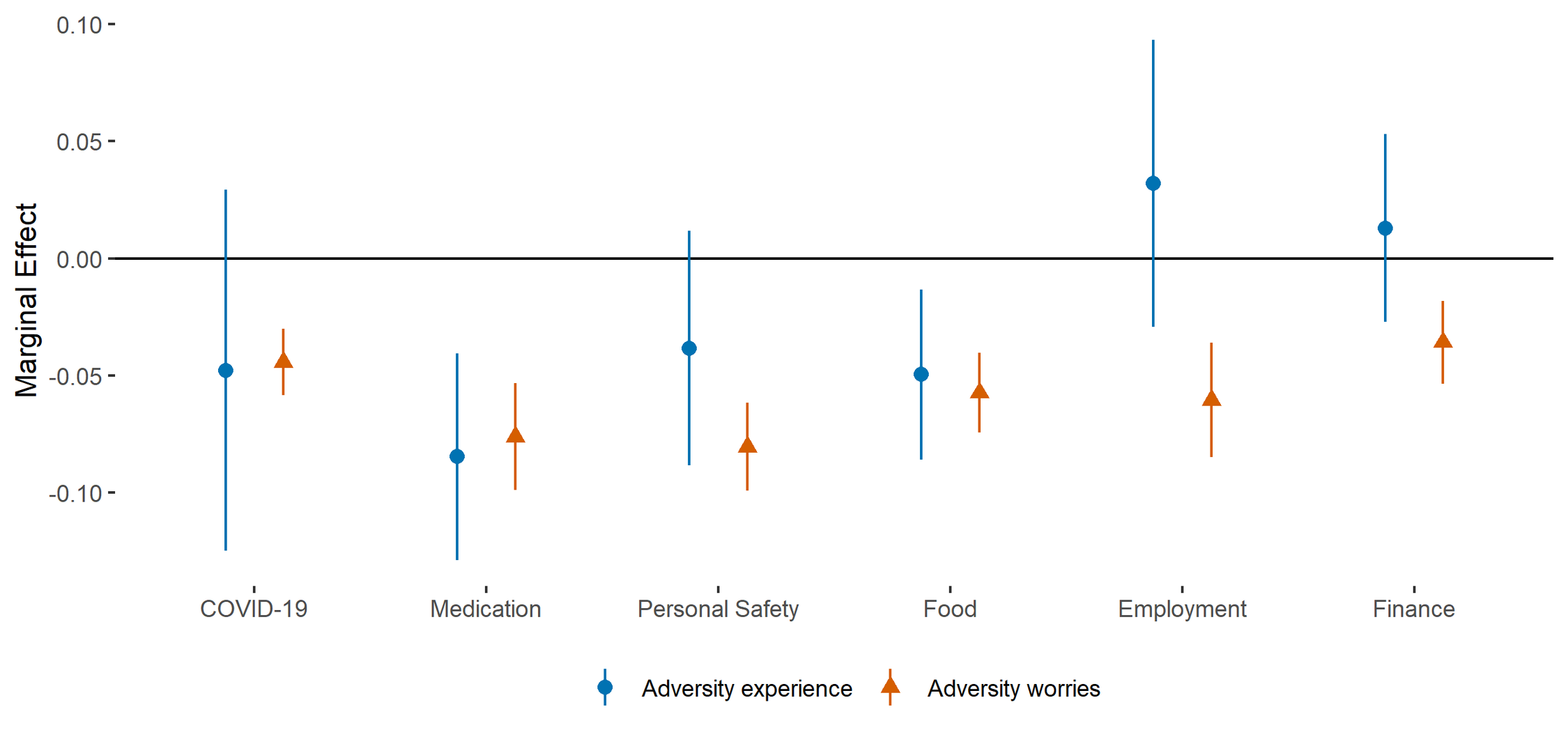

Supplement: S13 Fig — Derived from fixed effects models. (TIF) [file pone.0248919.s018.tif]

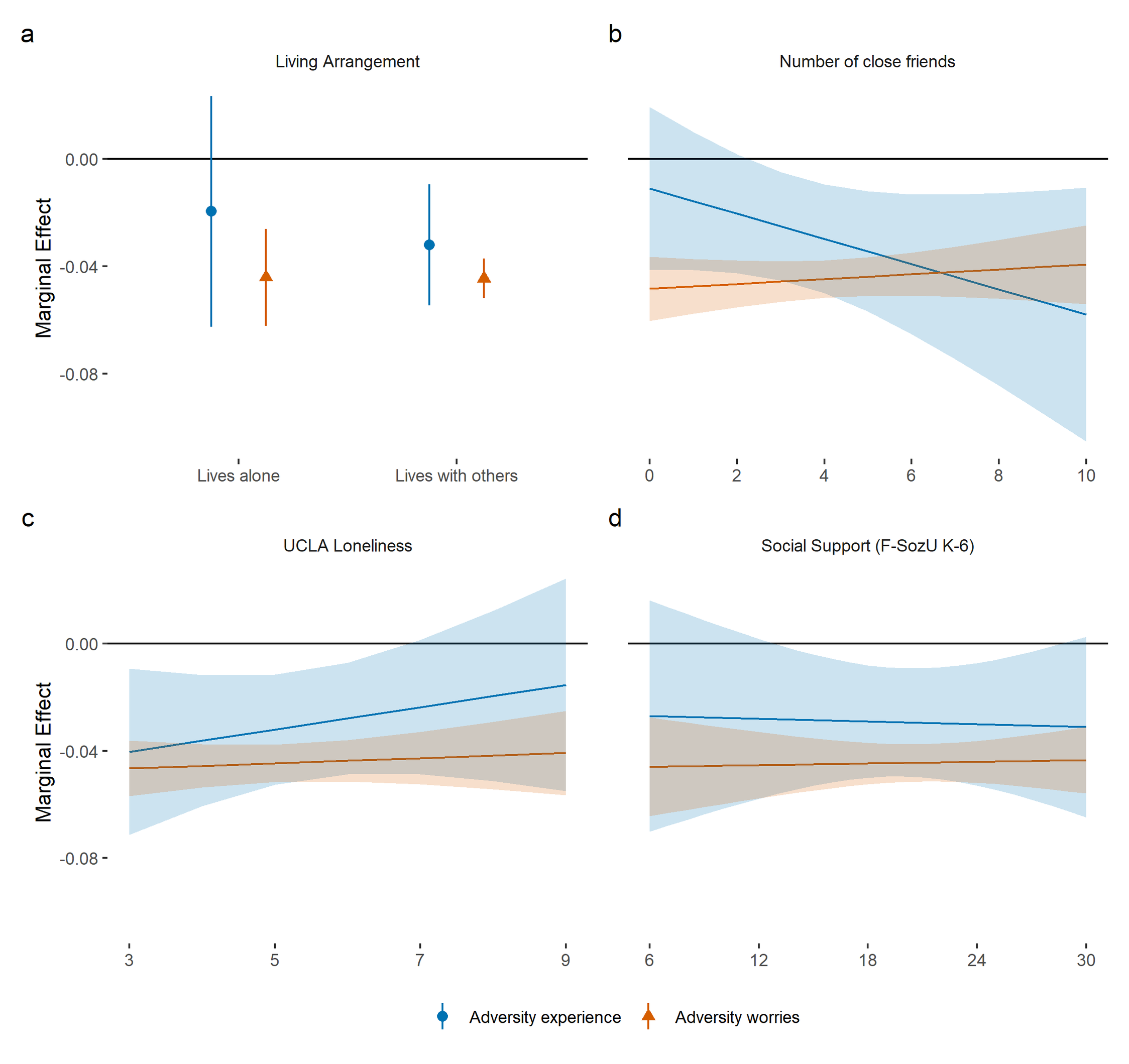

Supplement: S14 Fig — Derived from fixed effects models. (TIF) [file pone.0248919.s019.tif]

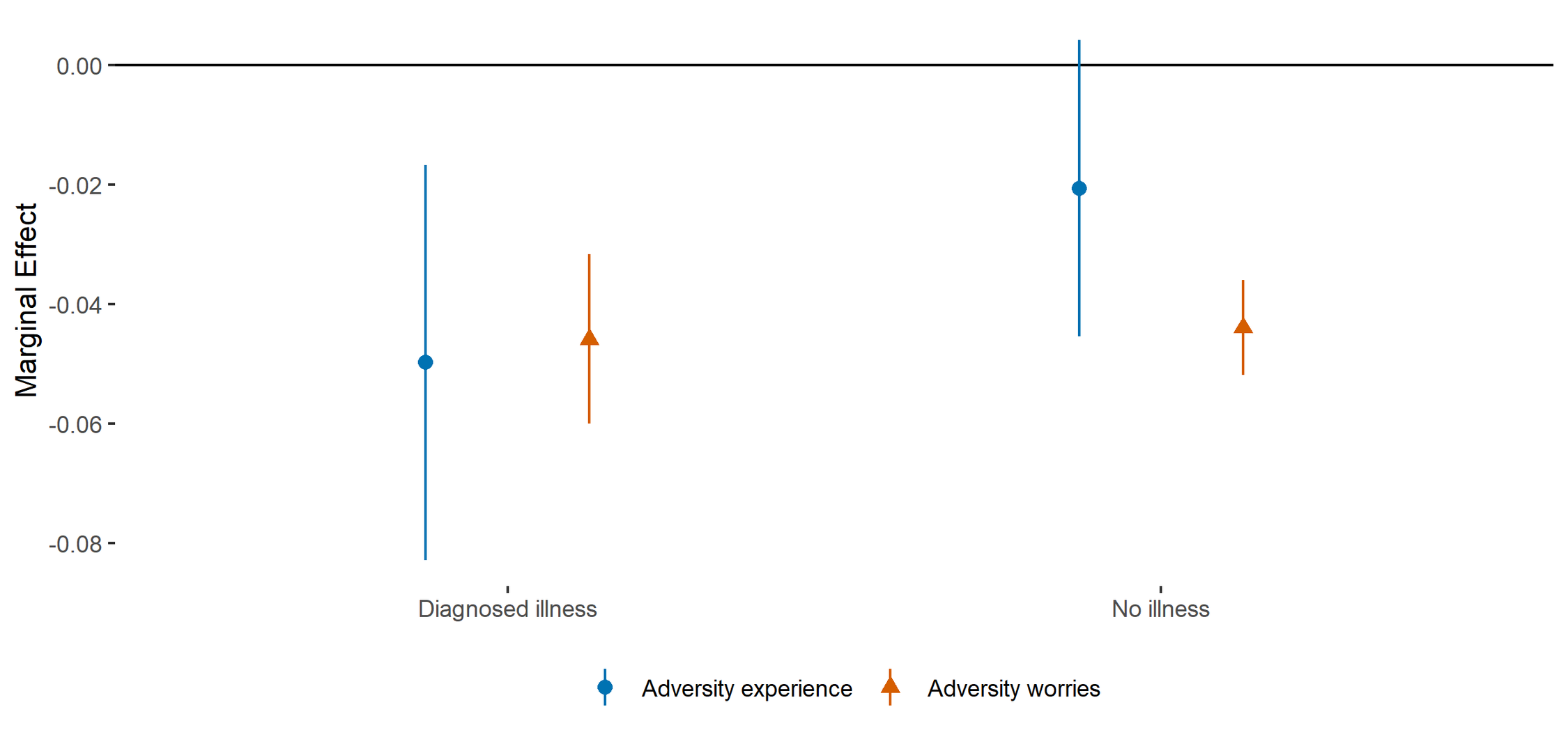

Supplement: S15 Fig — Derived from fixed effects models. (TIF) [file pone.0248919.s020.tif]

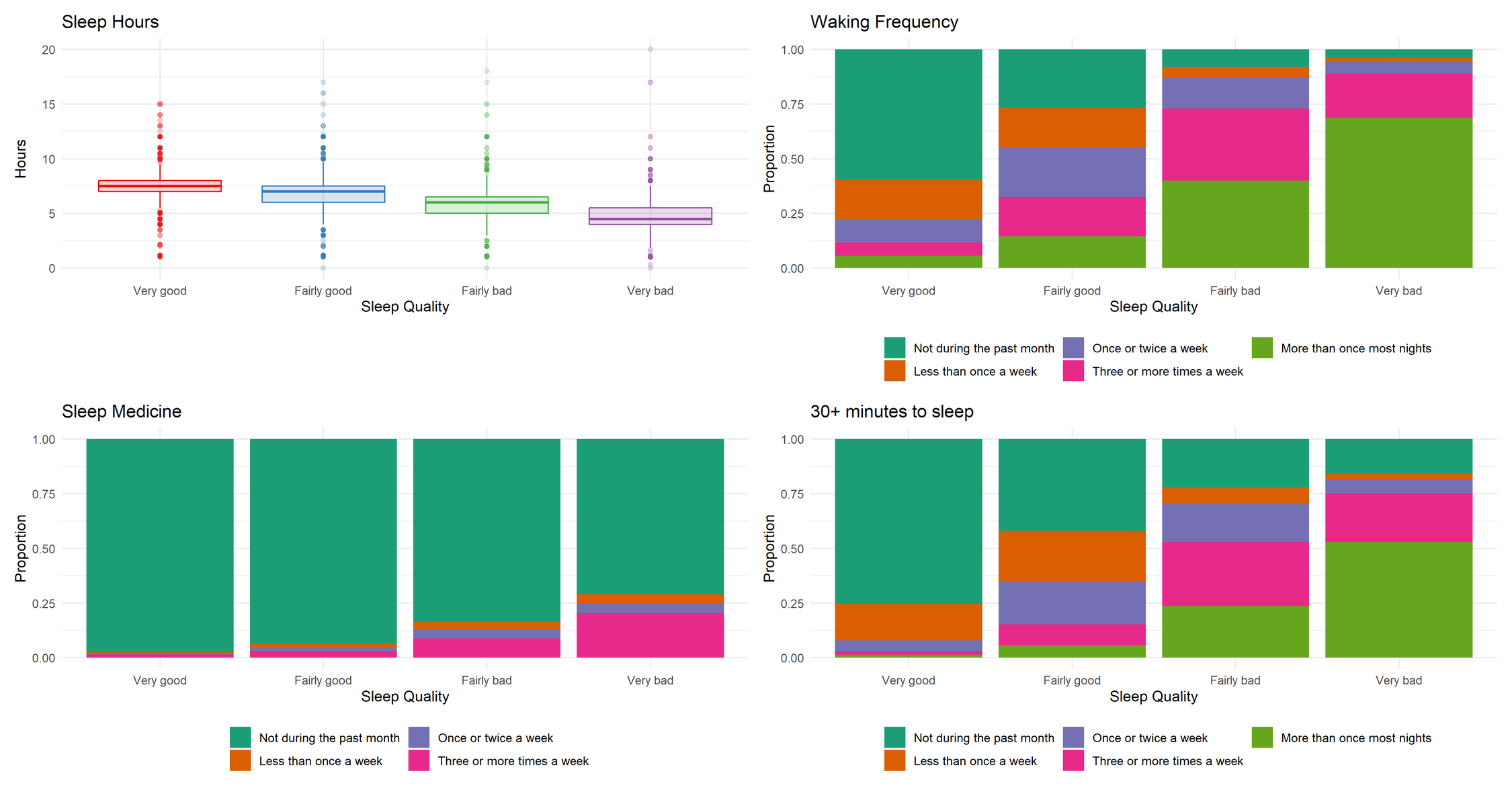

Supplement: S16 Fig — Sleep quality: “During the past month, how would you rate your sleep quality overall?”. Sleep hours: “How many hours of actual sleep did you usually get per night during the last month?”. 30+ minutes to sleep: “During the past month, how often have you had trouble sleeping because you… cannot get to sleep within 30 minutes?”. Waking frequency: “(During the past month, how often have you had trouble sleeping because you…) wake up in the middle of the night or early in the morning?”. Sleep medicine: “During the past month, how often have you taken medicine (prescribed or "over the counter") to help you sleep?”. The correlation between sleep quality and sleep time is 0.51. (TIF) [file pone.0248919.s021.tif]
