# Supplementary material for: Are adversities and worries during the COVID-19 pandemic related to sleep quality? Longitudinal analyses of 46,000 UK adults
Source: PLoS One. 2021 Mar 25;16(3):e0248919. doi: 10.1371/journal.pone.0248919 (PMC7993810; doi:10.1371/journal.pone.0248919)
Supplement: Attachment — Submitted filename: renamed_aaf3b.docx [file pone.0248919.s023.docx]

**COMMENTS – Manuscript PONE-D-20-32170**

Title: “Are adversities and worries during the COVID-19 pandemic related to sleep quality? Longitudinal analyses of 46,000 UK adults”

**Important note: This review pertains only to ‘statistical aspects’ of the study and so** **‘****clinical aspects’ [like medical importance,** **relevance of the study,** **‘clinical significance and** **implication(s)’ of the whole study, etc.] are to be evaluated [should be assessed] separately/independently. Further please note that any ‘statistical review’ is generally done under the assumption that (such) study specific methodological [as well as execution] issues are perfectly taken care of by the investigator(s). This review is not an exception to that and so does not cover clinical aspects {however, seldom comments are made only if those issues are intimately / scientifically related & intermingle with ‘statistical aspects’ of the study}. Agreed that ‘statistical methods’ are used as just tools here, however, they are vital part of methodology [and so should be given due importance].**

**COMMENTS:** Your ABSTRACT is well drafted but assay type. Please note that it is preferable [refer to item 1b of CONSORT checklist 2010: Structured summary of trial design, methods, results, and conclusions] to divide the ABSTRACT with small sections like ‘Objective(s)’, ‘Methods’, ‘Results’, ‘Conclusions’, etc. which is an accepted practice of most good/standard journals [including PLOS-ONE]. It will definitely be more informative then, I guess, whatever the article type may be.

I find lot many things common in this article with that of reference number 28 [Wright L, Steptoe A, Fancourt D. How are adversities during COVID-19 affecting mental health? Differential associations for worries and experiences and implications for policy. medRxiv. 2020 May 19;2020.05.14.20101717] given here (though the focus now is on pathway through effects on quality of sleep). It is natural as the data-base is probably same and the authors are same (in same sequence), however (though that is alright), some comments on or summary of overlap is desirable to appear in this article in brief. Although the topic [Are adversities and worries during the COVID-19 pandemic related to sleep quality?] dealt in this article is interesting, at least few things, if not many, here are ‘obvious’ in my opinion.

What exactly you mean by “The study is not random but does contain a well-stratified sample” (lines 93-4)? [no stratified sampling done then is this conclusion/finding/observation based on post-stratification?] Rest of the methodology and statistical methods used are correct [very good]. It is appreciated that ‘logit’ is used in hybrid models {though ‘logit’ is the only appropriate choice, often mistakenly ‘linear’ model(s) are used}.

Nothing more could be suggested/highlighted.
