# Supplementary material for: Are adversities and worries during the COVID-19 pandemic related to sleep quality? Longitudinal analyses of 46,000 UK adults
Source: PLoS One. 2021 Mar 25;16(3):e0248919. doi: 10.1371/journal.pone.0248919 (PMC7993810; doi:10.1371/journal.pone.0248919)
Supplement: Attachment — Submitted filename: reviewer_comments_2020_01_18.docx [file pone.0248919.s024.docx]

Dear Dr Vickers,

We hope you had a restful Christmas break.

Thank you to you and the reviewers for accepting our work for publication with revisions. We have uploaded an amended manuscript based on your comments. In the table below we respond to each of the points raised directly.

Best wishes,

Daisy

**Editor**

| Comment | Response |
| --- | --- |
| Please ensure that your manuscript meets PLOS ONE's style requirements, including those for file naming | The files are now in PLOS One’s house format. |
| Please provide the following information in your Methods section relating to the inter-views conducted in the study. In this case, please detail 1) the training and background of the inter-viewer, 2) where the interviews took place, 3) how long each interview lasted. | We apologise for our lack of clarity – we meant waves of data collection, rather than interviews. We have amended the text to reflect this. |
| We note that you have indicated that data from this study are available upon request. PLOS only allows data to be available upon request if there are legal or ethical restrictions on sharing data publicly. | The data will be made available at the end of 2021. This is due to stipulations set out by the ethics committee about the current political sensitivity of the data. However, we can provide data on request should someone contact us. |
| We note you have included a table to which you do not refer in the text of your manuscript. Please ensure that you refer to Table 2 in your text; if accepted, production will need this reference to link the reader to the Table. | We have rechecked the manuscript and every table we have referred to is included in the manuscript. |
| We noticed you have some minor occurrence of overlapping text with the following preprint, of which you are an author, which needs to be addressed: | We have altered text in the introduction and discussion to remove overlap. |

**Reviewer #1**

This manuscript examines how adverse events and worries affect sleep in these days of the COVID pandemic. The paper is obviously timely, and the large stratified sample (~46,000) from the UK is a strength of the study. Both worries and adverse events had a small deleterious effect on sleep, with worries being slightly more impactful. Interestingly, social network had relatively little impact on this effect.

| Comment | Response |
| --- | --- |
| The paper is heavy on statistical analysis and light on discussion. A different reviewer would be better to comment on that statistical analysis. | We have now added some more text to the discussion to expand on a few points. For example, we have added sentences tying our results on social support on other studies that have investigated the link between social support and sleep quality during the COVID-19 pandemic. This should help to unpack our findings more. |
| The authors have an overlapping paper (their reference number 28) which used the same sample in an effort to understand the impact of adversities and worries on mental health. Slicing their analyses so finely, makes it difficult to examine the interplay of sleep and mental health. | The papers are different as they have different outcomes and explore the role of other potential modifiers. However, the findings from the two papers do inform one another. We have now made more explicit reference to our other paper in the manuscript, noting that the analysis in the first paper inspired the analysis here in order to explore whether sleep is a biobehavioural pathway linking adversities with mental health outcomes. |
| There is a typo on Table 1. The rows “difficulties acquiring medication” and “difficulties accessing food” are reversed.  There is a glitch in the manuscript citation in line 1 of the demographics results section. | Thank you for noticing these errors. We have amended both.  Also, in the previous version of the manuscript, we reported confidence intervals without scale the sample weights. This has now been amended. |
| It is intriguing that so few people were worried about “catching COVID-19” (see table following line 210). That sample would be interesting in its own right. What sorts of life situations contribute to such worries or conversely make some people oblivious to the worries? | Yes, this is interesting. We have included some analysis in the demographics section of the results (also see S1 Fig). Among other factors, males, young people, people without partners, and those with high income and university education were less worried about catching the virus on average. |
| I am not sure that the figures add that much value to the paper. | We disagree with this. While the results could have been reported in tables or solely in the text, we believe that visualizations make the results much easier to perceive and remember. Some results are also only possible to show visually – for instance, drawing the results in Fig 3 is necessary to understand how confidence intervals change non-linearly across the range on the measures of social support. We’d also add that visualizations allow the reader to make a number of queries that we might not have thought of – for instance, to quickly assess the relative magnitude of differences at different levels of the social support modifiers. |

**Reviewer #2**

| Comment | Response |
| --- | --- |
| Your ABSTRACT is well drafted but assay type. Please note that it is preferable [refer to item 1b of CONSORT checklist 2010: Structured summary of trial design, methods, results, and conclusions] to divide the ABSTRACT with small sections like ‘Objective(s)’, ‘Methods’, ‘Results’, ‘Conclusions’, etc. which is an accepted practice of most good/standard journals [including PLOS-ONE]. | We have now added section titles to the abstract. |
| I find lot many things common in this article with that of reference number 28 [Wright L, Steptoe A, Fancourt D. How are adversities during COVID-19 affecting mental health? Differential associations for worries and experiences and implications for policy. medRxiv. 2020 May 19;2020.05.14.20101717] given here (though the focus now is on pathway through effects on quality of sleep). It is natural as the data-base is probably same and the authors are same (in same sequence), however (though that is alright), some comments on or summary of overlap is desirable to appear in this article in brief. | Please see our response to Reviewer #1 where we explain the distinctions more clearly. Also, we have rewritten sections of the introduction and discussion to remove overlap between the papers. |
| What exactly you mean by “The study is not random but does contain a well-stratified sample” (lines 93-4)? [no stratified sampling done then is this conclusion/finding/observation based on post-stratification?] | We are sorry this is unclear. We meant that the sample was heterogeneous in the demographics represented. We have now replaced “well-stratified” with “heterogeneous”. |
