# Supplementary material for: Are adversities and worries during the COVID-19 pandemic related to sleep quality? Longitudinal analyses of 46,000 UK adults
Source: PLoS One. 2021 Mar 25;16(3):e0248919. doi: 10.1371/journal.pone.0248919 (PMC7993810; doi:10.1371/journal.pone.0248919)
Supplement: Attachment — Submitted filename: reviewer_comments_2021_02_18.docx [file pone.0248919.s025.docx]

Dear Dr Vickers,

Thank you to you and the reviewers for accepting our work for publication with revisions. We have uploaded an amended manuscript based on your comments. Below we respond to each of the points raised directly.

Best wishes,

Daisy

**Editor**

Also, please consider whether you can expand on how this manuscript is related to the pre-print (now accepted for publication; congratulations). I see one sentence that references your prior work (reference 28). “Specifically during COVID-19, we have shown in recent work that there is a relationship between worries and experiences of adversities and poorer mental health [28].” Please consider if there is other information you could add to clarify how these studies are related (was the same sample used, for example; were the 41,909 UK adults in the pre-print also included in the 46,284 participants in this work). I look forward to receiving your minor revision of this manuscript.

Response:

Thank you. We’ve now added two sentences on how the samples overlap (we use complete case data in both studies, so the differences are due to small differences in variables used in the two studies). We’ve also updated the reference to the published version of the previous study.

**Reviewer #2**

Please note that the meaning of the terms “well stratified” and “heterogeneous” are not same Therefore, your response/answer to my comment/question regarding stratification that “We meant that the sample was heterogeneous in the demographics represented” is not acceptable {and even liked} as what you meant cannot be understood [how do expect that?] automatically. Remember that this is a scientific/academic document and so all details should be accurately/clearly/correctly communicated, and terms used carefully. Agreed that English is not my mother tongue however, I never knew that the term “well stratified” is alternative to “heterogeneous”.

Response:

We apologise again for the mistake. It was unintentional and we are glad it has now been rectified.
